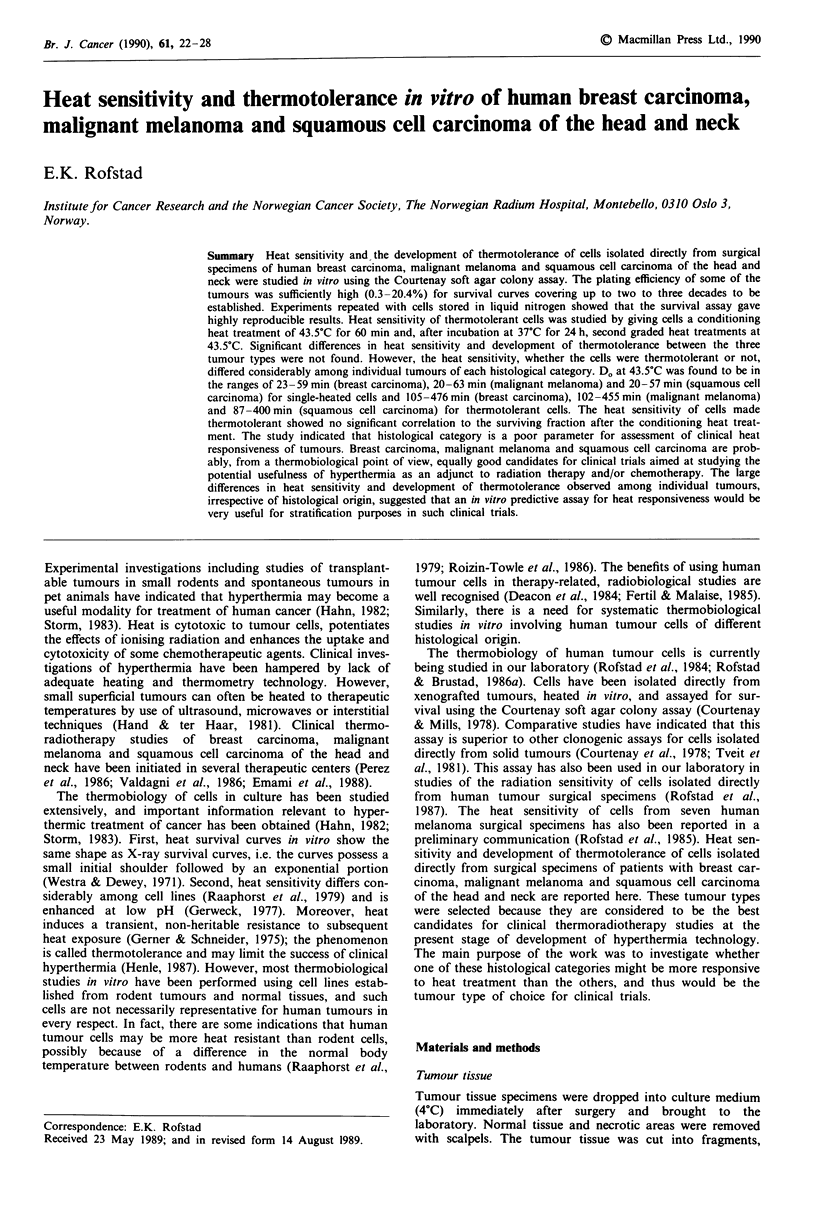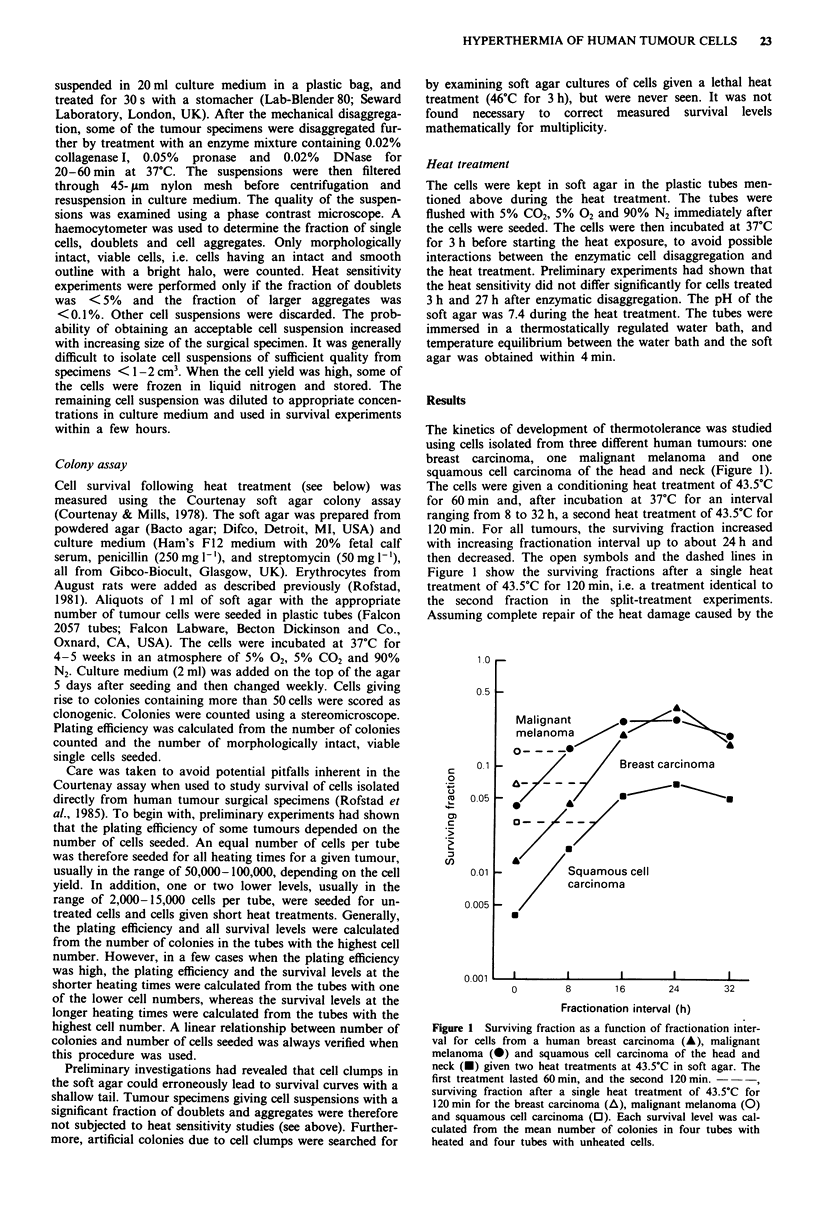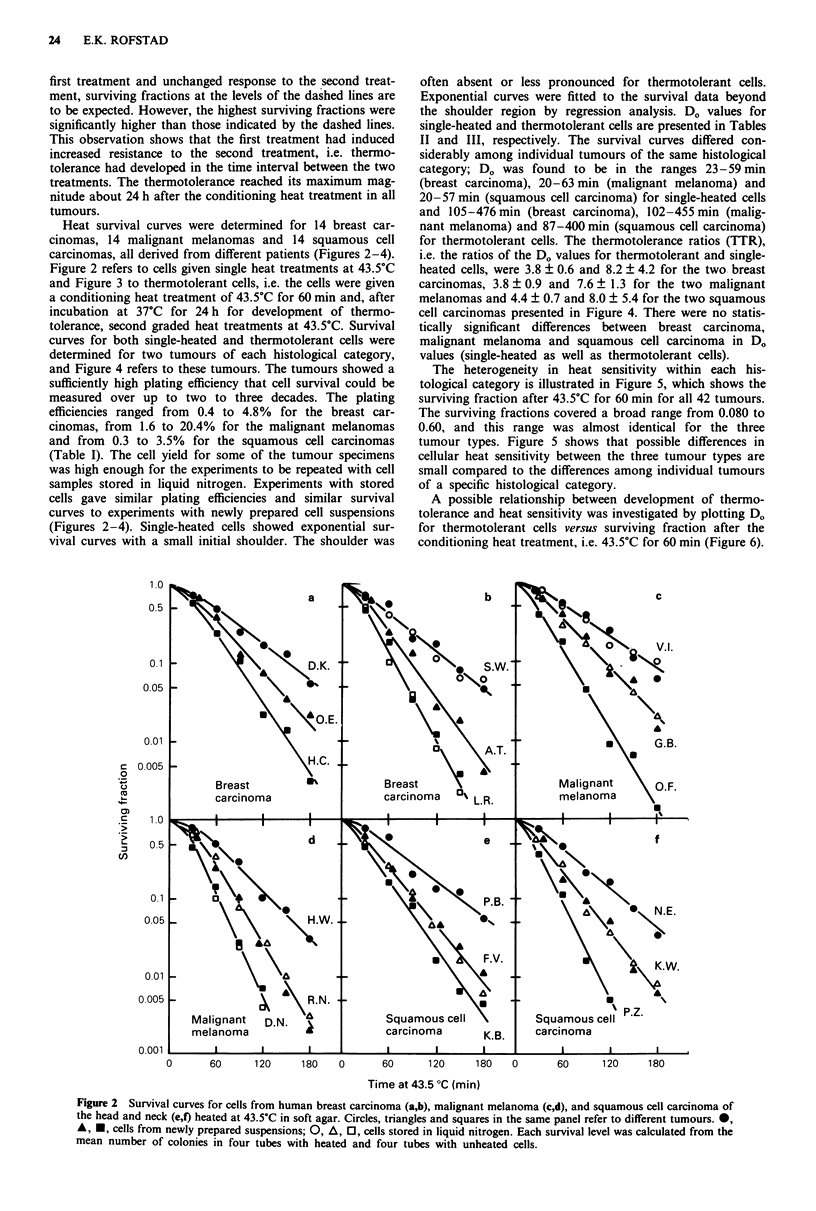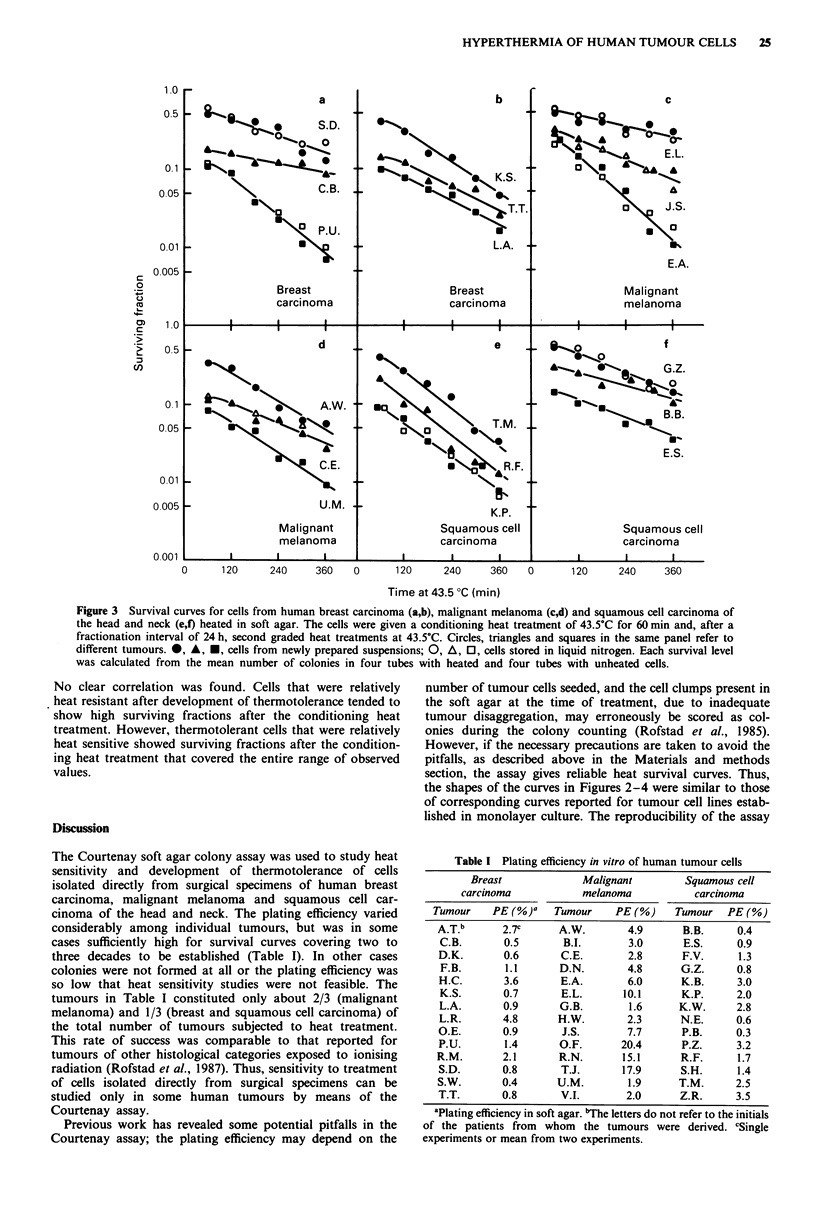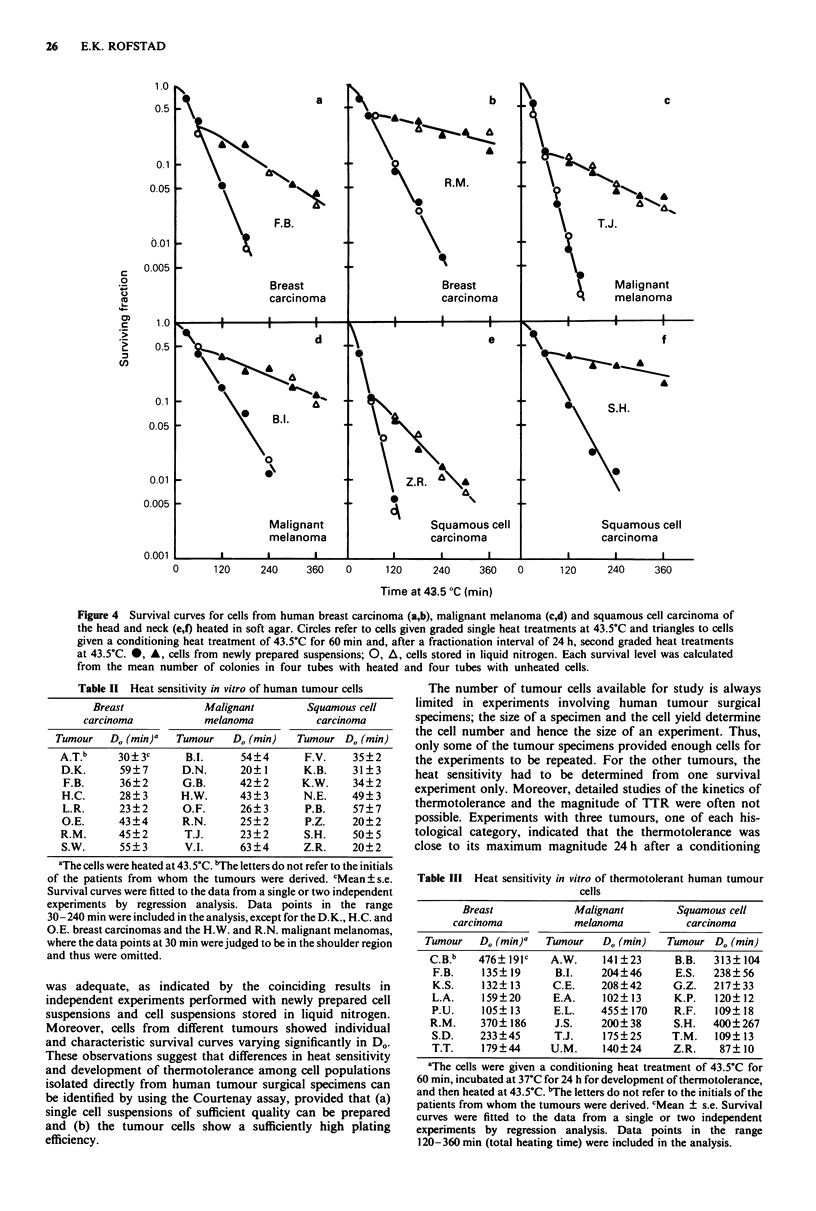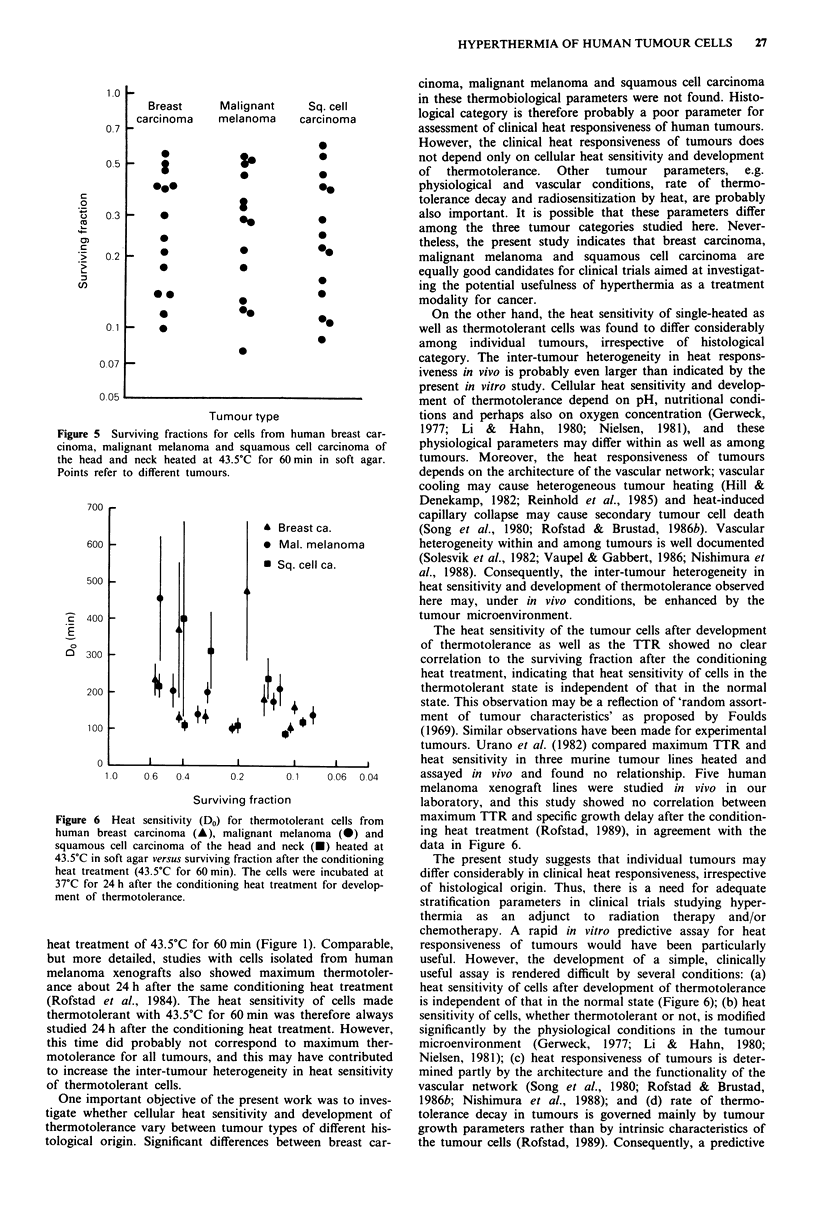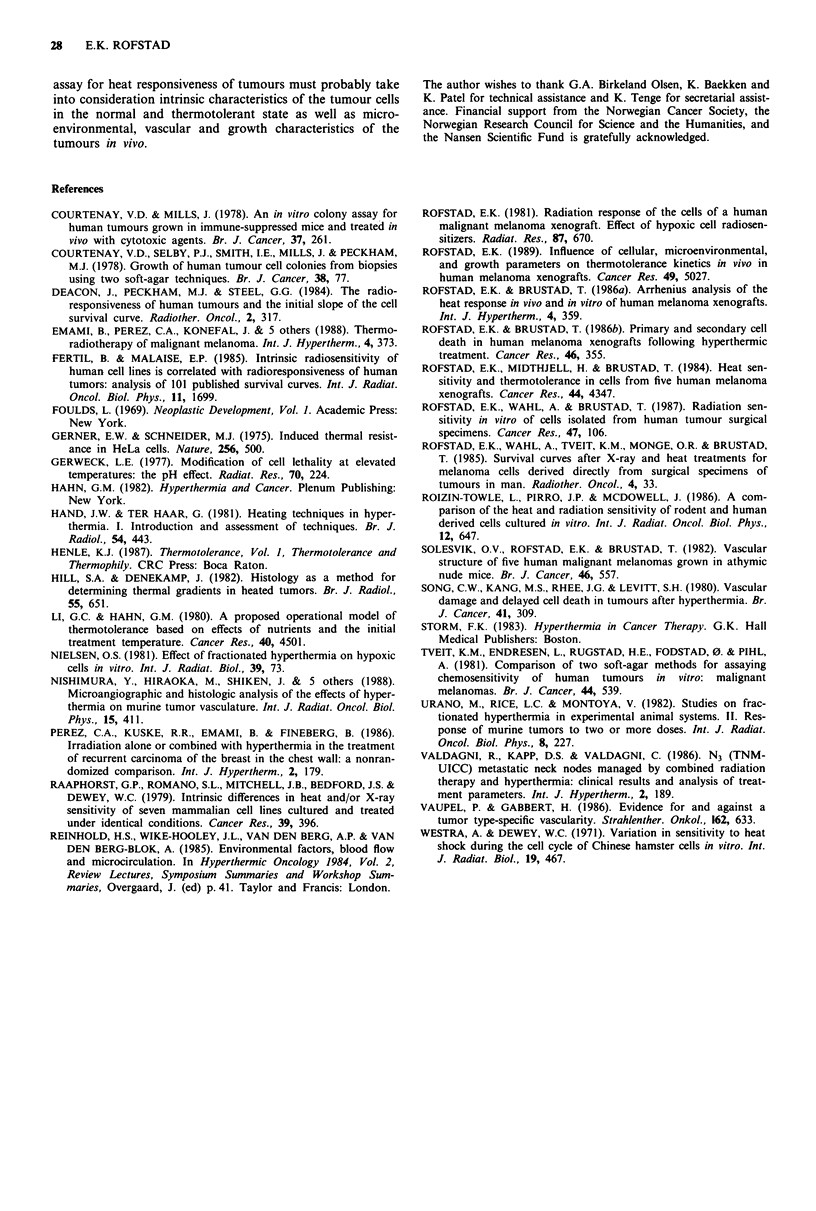# Heat sensitivity and thermotolerance in vitro of human breast carcinoma, malignant melanoma and squamous cell carcinoma of the head and neck.

**DOI:** 10.1038/bjc.1990.6

**Published:** 1990-01

**Authors:** E. K. Rofstad

**Affiliations:** Institute for Cancer Research, Norwegian Radium Hospital, Montebello.

## Abstract

Heat sensitivity and the development of thermotolerance of cells isolated directly from surgical specimens of human breast carcinoma, malignant melanoma and squamous cell carcinoma of the head and neck were studied in vitro using the Courtenay soft agar colony assay. The plating efficiency of some of the tumours was sufficiently high (0.3-20.4%) for survival curves covering up to two to three decades to be established. Experiments repeated with cells stored in liquid nitrogen showed that the survival assay gave highly reproducible results. Heat sensitivity of thermotolerant cells was studied by giving cells a conditioning heat treatment of 43.5 degrees C for 60 min and, after incubation at 37 degrees C for 24 h, second graded heat treatments at 43.5 degrees C. Significant differences in heat sensitivity and development of thermotolerance between the three tumour types were not found. However, the heat sensitivity, whether the cells were thermotolerant or not, differed considerably among individual tumours of each histological category. Do at 43.5 degrees C was found to be in the ranges of 23-59 min (breast carcinoma), 20-63 min (malignant melanoma) and 20-57 min (squamous cell carcinoma) for single-heated cells and 105-476 min (breast carcinoma). 102-455 min (malignant melanoma) and 87-400 min (squamous cell carcinoma) for thermotolerant cells. The heat sensitivity of cells made thermotolerant showed no significant correlation to the surviving fraction after the conditioning heat treatment. The study indicated that histological category is a poor parameter for assessment of clinical heat responsiveness of tumours. Breast carcinoma, malignant melanoma and squamous cell carcinoma are probably, from a thermobiological point of view, equally good candidates for clinical trial aimed at studying the potential usefulness of hyperthermia as an adjunct to radiation therapy and/or chemotherapy. The large differences in heat sensitivity and development of thermotolerance observed among individual tumours, irrespective of histological origin, suggested that an in vitro predictive assay for heat responsiveness would be very useful for stratification purposes in such clinical trials.


© Macmillan Press Ltd., 1990

### Heat sensitivity and thermotolerance *in vitro* of human breast carcinoma, malignant melanoma and squamous cell carcinoma of the head and neck

E.K. Rofstad

*Institute for Cancer Research and the Norwegian Cancer Society, The Norwegian Radium Hospital, Montebello, 0310 Oslo 3, Norway.*

**Summary** Heat sensitivity and the development of thermotolerance of cells isolated directly from surgical specimens of human breast carcinoma, malignant melanoma and squamous cell carcinoma of the head and neck were studied *in vitro* using the Courtenay soft agar colony assay. The plating efficiency of some of the tumours was sufficiently high (0.3-20.4%) for survival curves covering up to two to three decades to be established. Experiments repeated with cells stored in liquid nitrogen showed that the survival assay gave highly reproducible results. Heat sensitivity of thermotolerant cells was studied by giving cells a conditioning heat treatment of 43.5°C for 60 min and, after incubation at 37°C for 24 h, second graded heat treatments at 43.5°C. Significant differences in heat sensitivity and development of thermotolerance between the three tumour types were not found. However, the heat sensitivity, whether the cells were thermotolerant or not, differed considerably among individual tumours of each histological category. $D_0$ at 43.5°C was found to be in the ranges of 23-59 min (breast carcinoma), 20-63 min (malignant melanoma) and 20-57 min (squamous cell carcinoma) for single-heated cells and 105-476 min (breast carcinoma), 102-455 min (malignant melanoma) and 87-400 min (squamous cell carcinoma) for thermotolerant cells. The heat sensitivity of cells made thermotolerant showed no significant correlation to the surviving fraction after the conditioning heat treatment. The study indicated that histological category is a poor parameter for assessment of clinical heat responsiveness of tumours. Breast carcinoma, malignant melanoma and squamous cell carcinoma are probably, from a thermobiological point of view, equally good candidates for clinical trials aimed at studying the potential usefulness of hyperthermia as an adjunct to radiation therapy and/or chemotherapy. The large differences in heat sensitivity and development of thermotolerance observed among individual tumours, irrespective of histological origin, suggested that an *in vitro* predictive assay for heat responsiveness would be very useful for stratification purposes in such clinical trials.

Experimental investigations including studies of transplantable tumours in small rodents and spontaneous tumours in pet animals have indicated that hyperthermia may become a useful modality for treatment of human cancer (Hahn, 1982; Storm, 1983). Heat is cytotoxic to tumour cells, potentiates the effects of ionising radiation and enhances the uptake and cytotoxicity of some chemotherapeutic agents. Clinical investigations of hyperthermia have been hampered by lack of adequate heating and thermometry technology. However, small superficial tumours can often be heated to therapeutic temperatures by use of ultrasound, microwaves or interstitial techniques (Hand & ter Haar, 1981). Clinical thermoradiotherapy studies of breast carcinoma, malignant melanoma and squamous cell carcinoma of the head and neck have been initiated in several therapeutic centers (Perez *et al.*, 1986; Valdagni *et al.*, 1986; Emami *et al.*, 1988).

The thermobiology of cells in culture has been studied extensively, and important information relevant to hyperthermic treatment of cancer has been obtained (Hahn, 1982; Storm, 1983). First, heat survival curves *in vitro* show the same shape as X-ray survival curves, i.e. the curves possess a small initial shoulder followed by an exponential portion (Westra & Dewey, 1971). Second, heat sensitivity differs considerably among cell lines (Raaphorst *et al.*, 1979) and is enhanced at low pH (Gerweck, 1977). Moreover, heat induces a transient, non-heritable resistance to subsequent heat exposure (Gerner & Schneider, 1975); the phenomenon is called thermotolerance and may limit the success of clinical hyperthermia (Henle, 1987). However, most thermobiological studies *in vitro* have been performed using cell lines established from rodent tumours and normal tissues, and such cells are not necessarily representative for human tumours in every respect. In fact, there are some indications that human tumour cells may be more heat resistant than rodent cells, possibly because of a difference in the normal body temperature between rodents and humans (Raaphorst *et al.*, 1979; Roizin-Towle *et al.*, 1986). The benefits of using human tumour cells in therapy-related, radiobiological studies are well recognised (Deacon *et al.*, 1984; Fertil & Malaise, 1985). Similarly, there is a need for systematic thermobiological studies *in vitro* involving human tumour cells of different histological origin.

The thermobiology of human tumour cells is currently being studied in our laboratory (Rofstad *et al.*, 1984; Rofstad & Brustad, 1986*a*). Cells have been isolated directly from xenografted tumours, heated *in vitro*, and assayed for survival using the Courtenay soft agar colony assay (Courtenay & Mills, 1978). Comparative studies have indicated that this assay is superior to other clonogenic assays for cells isolated directly from solid tumours (Courtenay *et al.*, 1978; Tveit *et al.*, 1981). This assay has also been used in our laboratory in studies of the radiation sensitivity of cells isolated directly from human tumour surgical specimens (Rofstad *et al.*, 1987). The heat sensitivity of cells from seven human melanoma surgical specimens has also been reported in a preliminary communication (Rofstad *et al.*, 1985). Heat sensitivity and development of thermotolerance of cells isolated directly from surgical specimens of patients with breast carcinoma, malignant melanoma and squamous cell carcinoma of the head and neck are reported here. These tumour types were selected because they are considered to be the best candidates for clinical thermoradiotherapy studies at the present stage of development of hyperthermia technology. The main purpose of the work was to investigate whether one of these histological categories might be more responsive to heat treatment than the others, and thus would be the tumour type of choice for clinical trials.

### Materials and methods

### *Tumour tissue*

Tumour tissue specimens were dropped into culture medium (4°C) immediately after surgery and brought to the laboratory. Normal tissue and necrotic areas were removed with scalpels. The tumour tissue was cut into fragments,

Correspondence: E.K. Rofstad
Received 23 May 1989; and in revised form 14 August 1989.

suspended in 20 ml culture medium in a plastic bag, and treated for 30 s with a stomacher (Lab-Blender 80; Seward Laboratory, London, UK). After the mechanical disaggregation, some of the tumour specimens were disaggregated further by treatment with an enzyme mixture containing 0.02% collagenase I, 0.05% pronase and 0.02% DNase for 20–60 min at 37°C. The suspensions were then filtered through 45-μm nylon mesh before centrifugation and resuspension in culture medium. The quality of the suspensions was examined using a phase contrast microscope. A haemocytometer was used to determine the fraction of single cells, doublets and cell aggregates. Only morphologically intact, viable cells, i.e. cells having an intact and smooth outline with a bright halo, were counted. Heat sensitivity experiments were performed only if the fraction of doublets was <5% and the fraction of larger aggregates was <0.1%. Other cell suspensions were discarded. The probability of obtaining an acceptable cell suspension increased with increasing size of the surgical specimen. It was generally difficult to isolate cell suspensions of sufficient quality from specimens <1–2 $cm^3$. When the cell yield was high, some of the cells were frozen in liquid nitrogen and stored. The remaining cell suspension was diluted to appropriate concentrations in culture medium and used in survival experiments within a few hours.

*Colony assay*

Cell survival following heat treatment (see below) was measured using the Courtenay soft agar colony assay (Courtenay & Mills, 1978). The soft agar was prepared from powdered agar (Bacto agar; Difco, Detroit, MI, USA) and culture medium (Ham's F12 medium with 20% fetal calf serum, penicillin (250 mg $l^{-1}$), and streptomycin (50 mg $l^{-1}$), all from Gibco-Biocult, Glasgow, UK). Erythrocytes from August rats were added as described previously (Rofstad, 1981). Aliquots of 1 ml of soft agar with the appropriate number of tumour cells were seeded in plastic tubes (Falcon 2057 tubes; Falcon Labware, Becton Dickinson and Co., Oxnard, CA, USA). The cells were incubated at 37°C for 4–5 weeks in an atmosphere of 5% $O_2$, 5% $CO_2$ and 90% $N_2$. Culture medium (2 ml) was added on the top of the agar 5 days after seeding and then changed weekly. Cells giving rise to colonies containing more than 50 cells were scored as clonogenic. Colonies were counted using a stereomicroscope. Plating efficiency was calculated from the number of colonies counted and the number of morphologically intact, viable single cells seeded.

Care was taken to avoid potential pitfalls inherent in the Courtenay assay when used to study survival of cells isolated directly from human tumour surgical specimens (Rofstad *et al.*, 1985). To begin with, preliminary experiments had shown that the plating efficiency of some tumours depended on the number of cells seeded. An equal number of cells per tube was therefore seeded for all heating times for a given tumour, usually in the range of 50,000–100,000, depending on the cell yield. In addition, one or two lower levels, usually in the range of 2,000–15,000 cells per tube, were seeded for untreated cells and cells given short heat treatments. Generally, the plating efficiency and all survival levels were calculated from the number of colonies in the tubes with the highest cell number. However, in a few cases when the plating efficiency was high, the plating efficiency and the survival levels at the shorter heating times were calculated from the tubes with one of the lower cell numbers, whereas the survival levels at the longer heating times were calculated from the tubes with the highest cell number. A linear relationship between number of colonies and number of cells seeded was always verified when this procedure was used.

Preliminary investigations had revealed that cell clumps in the soft agar could erroneously lead to survival curves with a shallow tail. Tumour specimens giving cell suspensions with a significant fraction of doublets and aggregates were therefore not subjected to heat sensitivity studies (see above). Furthermore, artificial colonies due to cell clumps were searched for by examining soft agar cultures of cells given a lethal heat treatment (46°C for 3 h), but were never seen. It was not found necessary to correct measured survival levels mathematically for multiplicity.

*Heat treatment*

The cells were kept in soft agar in the plastic tubes mentioned above during the heat treatment. The tubes were flushed with 5% $CO_2$, 5% $O_2$ and 90% $N_2$ immediately after the cells were seeded. The cells were then incubated at 37°C for 3 h before starting the heat exposure, to avoid possible interactions between the enzymatic cell disaggregation and the heat treatment. Preliminary experiments had shown that the heat sensitivity did not differ significantly for cells treated 3 h and 27 h after enzymatic disaggregation. The pH of the soft agar was 7.4 during the heat treatment. The tubes were immersed in a thermostatically regulated water bath, and temperature equilibrium between the water bath and the soft agar was obtained within 4 min.

### Results

The kinetics of development of thermotolerance was studied using cells isolated from three different human tumours: one breast carcinoma, one malignant melanoma and one squamous cell carcinoma of the head and neck (Figure 1). The cells were given a conditioning heat treatment of 43.5°C for 60 min and, after incubation at 37°C for an interval ranging from 8 to 32 h, a second heat treatment of 43.5°C for 120 min. For all tumours, the surviving fraction increased with increasing fractionation interval up to about 24 h and then decreased. The open symbols and the dashed lines in Figure 1 show the surviving fractions after a single heat treatment of 43.5°C for 120 min, i.e. a treatment identical to the second fraction in the split-treatment experiments. Assuming complete repair of the heat damage caused by the

**Figure 1** Surviving fraction as a function of fractionation interval for cells from a human breast carcinoma (▲), malignant melanoma (●) and squamous cell carcinoma of the head and neck (■) given two heat treatments at 43.5°C in soft agar. The first treatment lasted 60 min, and the second 120 min. — — —, surviving fraction after a single heat treatment of 43.5°C for 120 min for the breast carcinoma (△), malignant melanoma (○) and squamous cell carcinoma (□). Each survival level was calculated from the mean number of colonies in four tubes with heated and four tubes with unheated cells.

first treatment and unchanged response to the second treatment, surviving fractions at the levels of the dashed lines are to be expected. However, the highest surviving fractions were significantly higher than those indicated by the dashed lines. This observation shows that the first treatment had induced increased resistance to the second treatment, i.e. thermotolerance had developed in the time interval between the two treatments. The thermotolerance reached its maximum magnitude about 24 h after the conditioning heat treatment in all tumours.

Heat survival curves were determined for 14 breast carcinomas, 14 malignant melanomas and 14 squamous cell carcinomas, all derived from different patients (Figures 2–4). Figure 2 refers to cells given single heat treatments at 43.5°C and Figure 3 to thermotolerant cells, i.e. the cells were given a conditioning heat treatment of 43.5°C for 60 min and, after incubation at 37°C for 24 h for development of thermotolerance, second graded heat treatments at 43.5°C. Survival curves for both single-heated and thermotolerant cells were determined for two tumours of each histological category, and Figure 4 refers to these tumours. The tumours showed a sufficiently high plating efficiency that cell survival could be measured over up to two to three decades. The plating efficiencies ranged from 0.4 to 4.8% for the breast carcinomas, from 1.6 to 20.4% for the malignant melanomas and from 0.3 to 3.5% for the squamous cell carcinomas (Table I). The cell yield for some of the tumour specimens was high enough for the experiments to be repeated with cell samples stored in liquid nitrogen. Experiments with stored cells gave similar plating efficiencies and similar survival curves to experiments with newly prepared cell suspensions (Figures 2–4). Single-heated cells showed exponential survival curves with a small initial shoulder. The shoulder was often absent or less pronounced for thermotolerant cells. Exponential curves were fitted to the survival data beyond the shoulder region by regression analysis. $D_0$ values for single-heated and thermotolerant cells are presented in Tables II and III, respectively. The survival curves differed considerably among individual tumours of the same histological category; $D_0$ was found to be in the ranges 23–59 min (breast carcinoma), 20–63 min (malignant melanoma) and 20–57 min (squamous cell carcinoma) for single-heated cells and 105–476 min (breast carcinoma), 102–455 min (malignant melanoma) and 87–400 min (squamous cell carcinoma) for thermotolerant cells. The thermotolerance ratios (TTR), i.e. the ratios of the $D_0$ values for thermotolerant and single-heated cells, were $3.8 \pm 0.6$ and $8.2 \pm 4.2$ for the two breast carcinomas, $3.8 \pm 0.9$ and $7.6 \pm 1.3$ for the two malignant melanomas and $4.4 \pm 0.7$ and $8.0 \pm 5.4$ for the two squamous cell carcinomas presented in Figure 4. There were no statistically significant differences between breast carcinoma, malignant melanoma and squamous cell carcinoma in $D_0$ values (single-heated as well as thermotolerant cells).

The heterogeneity in heat sensitivity within each histological category is illustrated in Figure 5, which shows the surviving fraction after 43.5°C for 60 min for all 42 tumours. The surviving fractions covered a broad range from 0.080 to 0.60, and this range was almost identical for the three tumour types. Figure 5 shows that possible differences in cellular heat sensitivity between the three tumour types are small compared to the differences among individual tumours of a specific histological category.

A possible relationship between development of thermotolerance and heat sensitivity was investigated by plotting $D_0$ for thermotolerant cells *versus* surviving fraction after the conditioning heat treatment, i.e. 43.5°C for 60 min (Figure 6).

**Figure 2** Survival curves for cells from human breast carcinoma (**a**,**b**), malignant melanoma (**c**,**d**), and squamous cell carcinoma of the head and neck (**e**,**f**) heated at 43.5°C in soft agar. Circles, triangles and squares in the same panel refer to different tumours. ●, ▲, ■, cells from newly prepared suspensions; ○, △, □, cells stored in liquid nitrogen. Each survival level was calculated from the mean number of colonies in four tubes with heated and four tubes with unheated cells.

**Figure 3** Survival curves for cells from human breast carcinoma (**a**,**b**), malignant melanoma (**c**,**d**) and squamous cell carcinoma of the head and neck (**e**,**f**) heated in soft agar. The cells were given a conditioning heat treatment of 43.5°C for 60 min and, after a fractionation interval of 24 h, second graded heat treatments at 43.5°C. Circles, triangles and squares in the same panel refer to different tumours. ●, ▲, ■, cells from newly prepared suspensions; ○, △, □, cells stored in liquid nitrogen. Each survival level was calculated from the mean number of colonies in four tubes with heated and four tubes with unheated cells.

No clear correlation was found. Cells that were relatively heat resistant after development of thermotolerance tended to show high surviving fractions after the conditioning heat treatment. However, thermotolerant cells that were relatively heat sensitive showed surviving fractions after the conditioning heat treatment that covered the entire range of observed values.

### Discussion

The Courtenay soft agar colony assay was used to study heat sensitivity and development of thermotolerance of cells isolated directly from surgical specimens of human breast carcinoma, malignant melanoma and squamous cell carcinoma of the head and neck. The plating efficiency varied considerably among individual tumours, but was in some cases sufficiently high for survival curves covering two to three decades to be established (Table I). In other cases colonies were not formed at all or the plating efficiency was so low that heat sensitivity studies were not feasible. The tumours in Table I constituted only about 2/3 (malignant melanoma) and 1/3 (breast and squamous cell carcinoma) of the total number of tumours subjected to heat treatment. This rate of success was comparable to that reported for tumours of other histological categories exposed to ionising radiation (Rofstad *et al.*, 1987). Thus, sensitivity to treatment of cells isolated directly from surgical specimens can be studied only in some human tumours by means of the Courtenay assay.

Previous work has revealed some potential pitfalls in the Courtenay assay; the plating efficiency may depend on the number of tumour cells seeded, and the cell clumps present in the soft agar at the time of treatment, due to inadequate tumour disaggregation, may erroneously be scored as colonies during the colony counting (Rofstad *et al.*, 1985). However, if the necessary precautions are taken to avoid the pitfalls, as described above in the Materials and methods section, the assay gives reliable heat survival curves. Thus, the shapes of the curves in Figures 2–4 were similar to those of corresponding curves reported for tumour cell lines established in monolayer culture. The reproducibility of the assay

**Table I** Plating efficiency *in vitro* of human tumour cells

| *Breast carcinoma* | | *Malignant melanoma* | | *Squamous cell carcinoma* | |
|---|---|---|---|---|---|
| *Tumour* | *PE (%)*[a] | *Tumour* | *PE (%)* | *Tumour* | *PE (%)* |
| A.T.[b] | 2.7[c] | A.W. | 4.9 | B.B. | 0.4 |
| C.B. | 0.5 | B.I. | 3.0 | E.S. | 0.9 |
| D.K. | 0.6 | C.E. | 2.8 | F.V. | 1.3 |
| F.B. | 1.1 | D.N. | 4.8 | G.Z. | 0.8 |
| H.C. | 3.6 | E.A. | 6.0 | K.B. | 3.0 |
| K.S. | 0.7 | E.L. | 10.1 | K.P. | 2.0 |
| L.A. | 0.9 | G.B. | 1.6 | K.W. | 2.8 |
| L.R. | 4.8 | H.W. | 2.3 | N.E. | 0.6 |
| O.E. | 0.9 | J.S. | 7.7 | P.B. | 0.3 |
| P.U. | 1.4 | O.F. | 20.4 | P.Z. | 3.2 |
| R.M. | 2.1 | R.N. | 15.1 | R.F. | 1.7 |
| S.D. | 0.8 | T.J. | 17.9 | S.H. | 1.4 |
| S.W. | 0.4 | U.M. | 1.9 | T.M. | 2.5 |
| T.T. | 0.8 | V.I. | 2.0 | Z.R. | 3.5 |

[a]Plating efficiency in soft agar. [b]The letters do not refer to the initials of the patients from whom the tumours were derived. [c]Single experiments or mean from two experiments.

**Figure 4** Survival curves for cells from human breast carcinoma (**a**,**b**), malignant melanoma (**c**,**d**) and squamous cell carcinoma of the head and neck (**e**,**f**) heated in soft agar. Circles refer to cells given graded single heat treatments at 43.5°C and triangles to cells given a conditioning heat treatment of 43.5°C for 60 min and, after a fractionation interval of 24 h, second graded heat treatments at 43.5°C. ●, ▲, cells from newly prepared suspensions; ○, △, cells stored in liquid nitrogen. Each survival level was calculated from the mean number of colonies in four tubes with heated and four tubes with unheated cells.

**Table II** Heat sensitivity *in vitro* of human tumour cells

| *Breast carcinoma* | | *Malignant melanoma* | | *Squamous cell carcinoma* | |
|---|---|---|---|---|---|
| *Tumour* | $D_o$ *(min)*[a] | *Tumour* | $D_o$ *(min)* | *Tumour* | $D_o$ *(min)* |
| A.T.[b] | 30±3[c] | B.I. | 54±4 | F.V. | 35±2 |
| D.K. | 59±7 | D.N. | 20±1 | K.B. | 31±3 |
| F.B. | 36±2 | G.B. | 42±2 | K.W. | 34±2 |
| H.C. | 28±3 | H.W. | 43±3 | N.E. | 49±3 |
| L.R. | 23±2 | O.F. | 26±3 | P.B. | 57±7 |
| O.E. | 43±4 | R.N. | 25±2 | P.Z. | 20±2 |
| R.M. | 45±2 | T.J. | 23±2 | S.H. | 50±5 |
| S.W. | 55±3 | V.I. | 63±4 | Z.R. | 20±2 |

[a]The cells were heated at 43.5°C. [b]The letters do not refer to the initials of the patients from whom the tumours were derived. [c]Mean±s.e. Survival curves were fitted to the data from a single or two independent experiments by regression analysis. Data points in the range 30–240 min were included in the analysis, except for the D.K., H.C. and O.E. breast carcinomas and the H.W. and R.N. malignant melanomas, where the data points at 30 min were judged to be in the shoulder region and thus were omitted.

was adequate, as indicated by the coinciding results in independent experiments performed with newly prepared cell suspensions and cell suspensions stored in liquid nitrogen. Moreover, cells from different tumours showed individual and characteristic survival curves varying significantly in $D_o$. These observations suggest that differences in heat sensitivity and development of thermotolerance among cell populations isolated directly from human tumour surgical specimens can be identified by using the Courtenay assay, provided that (a) single cell suspensions of sufficient quality can be prepared and (b) the tumour cells show a sufficiently high plating efficiency.

The number of tumour cells available for study is always limited in experiments involving human tumour surgical specimens; the size of a specimen and the cell yield determine the cell number and hence the size of an experiment. Thus, only some of the tumour specimens provided enough cells for the experiments to be repeated. For the other tumours, the heat sensitivity had to be determined from one survival experiment only. Moreover, detailed studies of the kinetics of thermotolerance and the magnitude of TTR were often not possible. Experiments with three tumours, one of each histological category, indicated that the thermotolerance was close to its maximum magnitude 24 h after a conditioning

**Table III** Heat sensitivity *in vitro* of thermotolerant human tumour cells

| *Breast carcinoma* | | *Malignant melanoma* | | *Squamous cell carcinoma* | |
|---|---|---|---|---|---|
| *Tumour* | $D_o$ *(min)*[a] | *Tumour* | $D_o$ *(min)* | *Tumour* | $D_o$ *(min)* |
| C.B.[b] | 476±191[c] | A.W. | 141±23 | B.B. | 313±104 |
| F.B. | 135±19 | B.I. | 204±46 | E.S. | 238±56 |
| K.S. | 132±13 | C.E. | 208±42 | G.Z. | 217±33 |
| L.A. | 159±20 | E.A. | 102±13 | K.P. | 120±12 |
| P.U. | 105±13 | E.L. | 455±170 | R.F. | 109±18 |
| R.M. | 370±186 | J.S. | 200±38 | S.H. | 400±267 |
| S.D. | 233±45 | T.J. | 175±25 | T.M. | 109±13 |
| T.T. | 179±44 | U.M. | 140±24 | Z.R. | 87±10 |

[a]The cells were given a conditioning heat treatment of 43.5°C for 60 min, incubated at 37°C for 24 h for development of thermotolerance, and then heated at 43.5°C. [b]The letters do not refer to the initials of the patients from whom the tumours were derived. [c]Mean ± s.e. Survival curves were fitted to the data from a single or two independent experiments by regression analysis. Data points in the range 120–360 min (total heating time) were included in the analysis.

**Figure 5** Surviving fractions for cells from human breast carcinoma, malignant melanoma and squamous cell carcinoma of the head and neck heated at 43.5°C for 60 min in soft agar. Points refer to different tumours.

**Figure 6** Heat sensitivity ($D_0$) for thermotolerant cells from human breast carcinoma (▲), malignant melanoma (●) and squamous cell carcinoma of the head and neck (■) heated at 43.5°C in soft agar *versus* surviving fraction after the conditioning heat treatment (43.5°C for 60 min). The cells were incubated at 37°C for 24 h after the conditioning heat treatment for development of thermotolerance.

heat treatment of 43.5°C for 60 min (Figure 1). Comparable, but more detailed, studies with cells isolated from human melanoma xenografts also showed maximum thermotolerance about 24 h after the same conditioning heat treatment (Rofstad *et al.*, 1984). The heat sensitivity of cells made thermotolerant with 43.5°C for 60 min was therefore always studied 24 h after the conditioning heat treatment. However, this time did probably not correspond to maximum thermotolerance for all tumours, and this may have contributed to increase the inter-tumour heterogeneity in heat sensitivity of thermotolerant cells.

One important objective of the present work was to investigate whether cellular heat sensitivity and development of thermotolerance vary between tumour types of different histological origin. Significant differences between breast carcinoma, malignant melanoma and squamous cell carcinoma in these thermobiological parameters were not found. Histological category is therefore probably a poor parameter for assessment of clinical heat responsiveness of human tumours. However, the clinical heat responsiveness of tumours does not depend only on cellular heat sensitivity and development of thermotolerance. Other tumour parameters, e.g. physiological and vascular conditions, rate of thermotolerance decay and radiosensitization by heat, are probably also important. It is possible that these parameters differ among the three tumour categories studied here. Nevertheless, the present study indicates that breast carcinoma, malignant melanoma and squamous cell carcinoma are equally good candidates for clinical trials aimed at investigating the potential usefulness of hyperthermia as a treatment modality for cancer.

On the other hand, the heat sensitivity of single-heated as well as thermotolerant cells was found to differ considerably among individual tumours, irrespective of histological category. The inter-tumour heterogeneity in heat responsiveness *in vivo* is probably even larger than indicated by the present *in vitro* study. Cellular heat sensitivity and development of thermotolerance depend on pH, nutritional conditions and perhaps also on oxygen concentration (Gerweck, 1977; Li & Hahn, 1980; Nielsen, 1981), and these physiological parameters may differ within as well as among tumours. Moreover, the heat responsiveness of tumours depends on the architecture of the vascular network; vascular cooling may cause heterogeneous tumour heating (Hill & Denekamp, 1982; Reinhold *et al.*, 1985) and heat-induced capillary collapse may cause secondary tumour cell death (Song *et al.*, 1980; Rofstad & Brustad, 1986*b*). Vascular heterogeneity within and among tumours is well documented (Solesvik *et al.*, 1982; Vaupel & Gabbert, 1986; Nishimura *et al.*, 1988). Consequently, the inter-tumour heterogeneity in heat sensitivity and development of thermotolerance observed here may, under *in vivo* conditions, be enhanced by the tumour microenvironment.

The heat sensitivity of the tumour cells after development of thermotolerance as well as the TTR showed no clear correlation to the surviving fraction after the conditioning heat treatment, indicating that heat sensitivity of cells in the thermotolerant state is independent of that in the normal state. This observation may be a reflection of 'random assortment of tumour characteristics' as proposed by Foulds (1969). Similar observations have been made for experimental tumours. Urano *et al.* (1982) compared maximum TTR and heat sensitivity in three murine tumour lines heated and assayed *in vivo* and found no relationship. Five human melanoma xenograft lines were studied *in vivo* in our laboratory, and this study showed no correlation between maximum TTR and specific growth delay after the conditioning heat treatment (Rofstad, 1989), in agreement with the data in Figure 6.

The present study suggests that individual tumours may differ considerably in clinical heat responsiveness, irrespective of histological origin. Thus, there is a need for adequate stratification parameters in clinical trials studying hyperthermia as an adjunct to radiation therapy and/or chemotherapy. A rapid *in vitro* predictive assay for heat responsiveness of tumours would have been particularly useful. However, the development of a simple, clinically useful assay is rendered difficult by several conditions: (a) heat sensitivity of cells after development of thermotolerance is independent of that in the normal state (Figure 6); (b) heat sensitivity of cells, whether thermotolerant or not, is modified significantly by the physiological conditions in the tumour microenvironment (Gerweck, 1977; Li & Hahn, 1980; Nielsen, 1981); (c) heat responsiveness of tumours is determined partly by the architecture and the functionality of the vascular network (Song *et al.*, 1980; Rofstad & Brustad, 1986*b*; Nishimura *et al.*, 1988); and (d) rate of thermotolerance decay in tumours is governed mainly by tumour growth parameters rather than by intrinsic characteristics of the tumour cells (Rofstad, 1989). Consequently, a predictive assay for heat responsiveness of tumours must probably take into consideration intrinsic characteristics of the tumour cells in the normal and thermotolerant state as well as microenvironmental, vascular and growth characteristics of the tumours *in vivo*.

The author wishes to thank G.A. Birkeland Olsen, K. Baekken and K. Patel for technical assistance and K. Tenge for secretarial assistance. Financial support from the Norwegian Cancer Society, the Norwegian Research Council for Science and the Humanities, and the Nansen Scientific Fund is gratefully acknowledged.

### References

COURTENAY, V.D. & MILLS, J. (1978). An *in vitro* colony assay for human tumours grown in immune-suppressed mice and treated *in vivo* with cytotoxic agents. *Br. J. Cancer*, **37**, 261.

COURTENAY, V.D., SELBY, P.J., SMITH, I.E., MILLS, J. & PECKHAM, M.J. (1978). Growth of human tumour cell colonies from biopsies using two soft-agar techniques. *Br. J. Cancer*, **38**, 77.

DEACON, J., PECKHAM, M.J. & STEEL, G.G. (1984). The radioresponsiveness of human tumours and the initial slope of the cell survival curve. *Radiother. Oncol.*, **2**, 317.

EMAMI, B., PEREZ, C.A., KONEFAL, J. & 5 others (1988). Thermoradiotherapy of malignant melanoma. *Int. J. Hypertherm.*, **4**, 373.

FERTIL, B. & MALAISE, E.P. (1985). Intrinsic radiosensitivity of human cell lines is correlated with radioresponsiveness of human tumors: analysis of 101 published survival curves. *Int. J. Radiat. Oncol. Biol. Phys.*, **11**, 1699.

FOULDS, L. (1969). *Neoplastic Development, Vol. 1*. Academic Press: New York.

GERNER, E.W. & SCHNEIDER, M.J. (1975). Induced thermal resistance in HeLa cells. *Nature*, **256**, 500.

GERWECK, L.E. (1977). Modification of cell lethality at elevated temperatures: the pH effect. *Radiat. Res.*, **70**, 224.

HAHN, G.M. (1982). *Hyperthermia and Cancer*. Plenum Publishing: New York.

HAND, J.W. & TER HAAR, G. (1981). Heating techniques in hyperthermia. I. Introduction and assessment of techniques. *Br. J. Radiol.*, **54**, 443.

HENLE, K.J. (1987). *Thermotolerance, Vol. 1, Thermotolerance and Thermophily*. CRC Press: Boca Raton.

HILL, S.A. & DENEKAMP, J. (1982). Histology as a method for determining thermal gradients in heated tumors. *Br. J. Radiol.*, **55**, 651.

LI, G.C. & HAHN, G.M. (1980). A proposed operational model of thermotolerance based on effects of nutrients and the initial treatment temperature. *Cancer Res.*, **40**, 4501.

NIELSEN, O.S. (1981). Effect of fractionated hyperthermia on hypoxic cells *in vitro*. *Int. J. Radiat. Biol.*, **39**, 73.

NISHIMURA, Y., HIRAOKA, M., SHIKEN, J. & 5 others (1988). Microangiographic and histologic analysis of the effects of hyperthermia on murine tumor vasculature. *Int. J. Radiat. Oncol. Biol. Phys.*, **15**, 411.

PEREZ, C.A., KUSKE, R.R., EMAMI, B. & FINEBERG, B. (1986). Irradiation alone or combined with hyperthermia in the treatment of recurrent carcinoma of the breast in the chest wall: a nonrandomized comparison. *Int. J. Hypertherm.*, **2**, 179.

RAAPHORST, G.P., ROMANO, S.L., MITCHELL, J.B., BEDFORD, J.S. & DEWEY, W.C. (1979). Intrinsic differences in heat and/or X-ray sensitivity of seven mammalian cell lines cultured and treated under identical conditions. *Cancer Res.*, **39**, 396.

REINHOLD, H.S., WIKE-HOOLEY, J.L., VAN DEN BERG, A.P. & VAN DEN BERG-BLOK, A. (1985). Environmental factors, blood flow and microcirculation. In *Hyperthermic Oncology 1984, Vol. 2, Review Lectures, Symposium Summaries and Workshop Summaries*, Overgaard, J. (ed) p. 41. Taylor and Francis: London.

ROFSTAD, E.K. (1981). Radiation response of the cells of a human malignant melanoma xenograft. Effect of hypoxic cell radiosensitizers. *Radiat. Res.*, **87**, 670.

ROFSTAD, E.K. (1989). Influence of cellular, microenvironmental, and growth parameters on thermotolerance kinetics *in vivo* in human melanoma xenografts. *Cancer Res.* **49**, 5027.

ROFSTAD, E.K. & BRUSTAD, T. (1986*a*). Arrhenius analysis of the heat response *in vivo* and *in vitro* of human melanoma xenografts. *Int. J. Hypertherm.*, **4**, 359.

ROFSTAD, E.K. & BRUSTAD, T. (1986*b*). Primary and secondary cell death in human melanoma xenografts following hyperthermic treatment. *Cancer Res.*, **46**, 355.

ROFSTAD, E.K., MIDTHJELL, H. & BRUSTAD, T. (1984). Heat sensitivity and thermotolerance in cells from five human melanoma xenografts. *Cancer Res.*, **44**, 4347.

ROFSTAD, E.K., WAHL, A. & BRUSTAD, T. (1987). Radiation sensitivity *in vitro* of cells isolated from human tumour surgical specimens. *Cancer Res.*, **47**, 106.

ROFSTAD, E.K., WAHL, A., TVEIT, K.M., MONGE, O.R. & BRUSTAD, T. (1985). Survival curves after X-ray and heat treatments for melanoma cells derived directly from surgical specimens of tumours in man. *Radiother. Oncol.*, **4**, 33.

ROIZIN-TOWLE, L., PIRRO, J.P. & MCDOWELL, J. (1986). A comparison of the heat and radiation sensitivity of rodent and human derived cells cultured *in vitro*. *Int. J. Radiat. Oncol. Biol. Phys.*, **12**, 647.

SOLESVIK, O.V., ROFSTAD, E.K. & BRUSTAD, T. (1982). Vascular structure of five human malignant melanomas grown in athymic nude mice. *Br. J. Cancer*, **46**, 557.

SONG, C.W., KANG, M.S., RHEE, J.G. & LEVITT, S.H. (1980). Vascular damage and delayed cell death in tumours after hyperthermia. *Br. J. Cancer*, **41**, 309.

STORM, F.K. (1983). *Hyperthermia in Cancer Therapy*. G.K. Hall Medical Publishers: Boston.

TVEIT, K.M., ENDRESEN, L., RUGSTAD, H.E., FODSTAD, Ø. & PIHL, A. (1981). Comparison of two soft-agar methods for assaying chemosensitivity of human tumours *in vitro*: malignant melanomas. *Br. J. Cancer*, **44**, 539.

URANO, M., RICE, L.C. & MONTOYA, V. (1982). Studies on fractionated hyperthermia in experimental animal systems. II. Response of murine tumors to two or more doses. *Int. J. Radiat. Oncol. Biol. Phys.*, **8**, 227.

VALDAGNI, R., KAPP, D.S. & VALDAGNI, C. (1986). $N_3$ (TNM-UICC) metastatic neck nodes managed by combined radiation therapy and hyperthermia: clinical results and analysis of treatment parameters. *Int. J. Hypertherm.*, **2**, 189.

VAUPEL, P. & GABBERT, H. (1986). Evidence for and against a tumor type-specific vascularity. *Strahlenther. Onkol.*, **162**, 633.

WESTRA, A. & DEWEY, W.C. (1971). Variation in sensitivity to heat shock during the cell cycle of Chinese hamster cells *in vitro*. *Int. J. Radiat. Biol.*, **19**, 467.